# Communication and Coordination Processes Supporting Integrated Transitional Care: Australian Healthcare Practitioners’ Perspectives

**DOI:** 10.5334/ijic.4685

**Published:** 2020-04-16

**Authors:** Jacqueline Allen, Alison M. Hutchinson, Rhonda Brown, Patricia M. Livingston

**Affiliations:** 1 School of Nursing and Midwifery, Monash University, Clayton, Vic, AU; 2 School of Nursing and Midwifery, Deakin University, Geelong, Vic, AU; 3 Centre for Quality and Patient Safety Research, Institute for Health Transformation, Deakin University, Geelong, Vic, AU

**Keywords:** integrated care, transitional care, communication, care coordination

## Abstract

**Introduction:**

Although a large body of research has identified effective models of transitional care, questions remain about the optimal translation of this knowledge into practice. In Australia, the introduction of a model of consumer-directed care uniquely challenges the practice of integrated care transitions for older adults. This study aimed to identify strengths and weaknesses in transitional care for older adults in an Australian setting by describing healthcare practitioners’ experiences of care provision.

**Methods:**

The study used a qualitative design in two phases: 1) semi-structured interviews, 2) one focus group. The setting comprised one public health network and five community services in urban Australia. In Phase 1, health practitioners across settings were interviewed about their experience of transitional care. Phase 2 sought feedback about the Phase 1 findings from different practitioners. All data were thematically analysed.

**Findings:**

In Phase 1), 48 healthcare practitioners were interviewed across multiple settings. Few participants were aware of the introduction of consumer-directed care in community aged care. Four main themes were identified: ‘Rapid and safe care transition’, ‘Discussing as a team’, ‘Questioning the discharge’, and ‘Engaging patients and carers’. In Phase 2), seven participants from different settings reviewed and endorsed the findings from Phase 1.

**Discussion and conclusions:**

Findings indicate that healthcare practitioners use a range of communication and coordination processes in optimising integrated transitional care. Although participants involved their patients in transitional care planning, most participants were unaware of the recent implementation of consumer-directed care. In contexts of community-based care shaped by multidisciplinary, sub-acute and CDC models, care integration must focus on improved communication with patients and carers to ascertain their needs and to support their increased responsibility in their care transitions.

## Introduction

Implementation of transitional care for older adults from hospital to home is challenging within healthcare contexts characterised by service fragmentation and increasing demand for aged care. Integrated transitional care is defined as coordinated and continuous care for patients across different health programs and settings [1]. Suboptimal care integration during care transitions results in unmet needs at home, unnecessary readmission to hospital and unwanted permanent placement in residential care [2]. In response to the need to improve transitional care for older adults, models of sub-acute care and advanced practice nursing are emerging in many western countries including the United Kingdom and North America [1,2]. This is resulting in stronger acute and sub-acute multidisciplinary models of care that work alongside specialty aged care teams [3]. Although a large body of research has identified effective models of transitional care that emphasise care integration [1,2], questions remain about the optimal translation of this knowledge into real-world practice settings.

### Integrated transitional care

Transitional care is referred to as continuous and unified care for patients across different health programs and settings [1]. Communication, care coordination, medication reconciliation, functional improvement and self-management are important features of transitional care [1,4]. In accordance with contemporary research [1,5,6,7], we focussed on communication and care coordination as essential processes in clinical care that support care integration during older adults’ care transitions.

Integrated transitional care for older adults with chronic illnesses is a focus of healthcare improvements in many Western countries [6,8,9]. In the UK, the National Health Service developed Health Trusts to improve care integration for patients, including transitional care, with an emphasis on enhanced communication and care planning between health practitioners in acute, sub-acute and community care, and on service efficiency [10]. Initiatives have also been implemented to improve transitional care for older adults in Europe [11] and the United States [12].

Previous studies have found that compared with usual care, formal transitional care interventions including discharge assessment, planning, care coordination, communication, medication reconciliation, and self-management reduce length of stay and re-admission rates, and improve patient satisfaction with care [7,13]. Two well-researched US-based models of care, the Care Transitions Intervention [4] and the Transitional Care Model [1] have been influential in re-orienting health services towards the importance of self-management and advanced practice nursing support. Other studies of transitional care have explored care integrated with multidisciplinary teams and aged care teams, and found reduced readmission rates and reduced functional decline in older adults [2,11].

### The Australian context

In Australia, health and aged care are characterised by service fragmentation [14]. Health and aged care are provided by multiple services and funded through varying state and federal government programs; for example, public hospital networks provide in-patient, outpatient, acute care, sub-acute care and some community-based services [14,15]. In Australia, the public health insurer, Medicare, funds or partially funds a range of health services including general practice (family medicine/physician) services [16].

In 2015 in Australia, community-aged care underwent major reforms with the implementation of a consumer-directed care (CDC) model and the Commonwealth Home Support Program [17,18]. In this model and program, older adults and their carers can access high-level aged care packages, and a range of personal and respite care services through local councils. Despite their many benefits, these reforms also resulted in de-implementation of publically funded community-based professional healthcare services such as district nursing without additional funding for healthcare professionals in general practice [19].

Despite improvements in knowledge due to previous research and practice initiatives, optimal implementation of integrated care, as supported by communication and care coordination, during older adults’ care transitions remains problematic in real-world practice [2,5]. In Australia, increasing service fragmentation and the introduction of the CDC model uniquely challenges the practice of care integration in older adults’ care transitions. Older adults and informal carers have greater responsibility in decision-making including navigating and negotiating their care transitions.

## Aim

The aim of this study was to identify implementation strengths and weaknesses in integrated transitional care for older adults in an Australian setting by describing how healthcare practitioners experience care provision across acute, sub-acute and community care programs.

## Theoretical Framework

This study is theoretically underpinned by social constructivism (constructivism). Constructivism is understood as social processes simultaneously creative of and created by people through interactions with their social context and with each other [20]. In this theoretical framework, experience is understood as relational to social context; therefore experience is socially constructed [20,21]. Social contexts in healthcare can include care approaches that are shaped and influenced by organisational and political imperatives such as those driving fast hospital throughput and early discharge. User experience in healthcare, including transitional care, is itself a social process [20,21]. User experience typically refers to the experience of patients. However, as healthcare practitioners apply transitional care interventions, in the current study they were recognized as users. The social process of user experience in transitional care formed the principal meaning unit for data analysis.

## Methods

A qualitative, exploratory design comprising two phases was used. Phase 1 focussed on ascertaining healthcare practitioners’ experiences of transitional care provision using semi-structured interviews. Phase 2 sought feedback about the Phase 1 findings from different practitioners, a patient and a carer using a focus group. In accordance with recommendations regarding qualitative health research [20,22], we included Phase 2 as part of the validation process to confirm findings from semi-structured interviews undertaken in Phase 1.

Healthcare practitioners were licensed to practice in their respective healthcare or allied healthcare profession in accordance with relevant Australian legislation and as certified by the Australian Health Practitioner Regulation Agency. Healthcare practitioners included registered nurses, enrolled nurses (licensed practical nurse), and medical practitioners and physicians. Allied health practitioners included physiotherapists (physical therapists), pharmacists, social workers, case managers and occupational therapists.

Both phases of the study were part of a larger study aiming to develop a communication tool for use by health practitioners, patients and caregivers [23,24]. As patients and caregivers were the end users, it was important to include their perspectives in the Phase 2 focus group regarding the communication tool that was developed. In this paper, health practitioners’ perspectives are presented.

### Setting

As health and aged care are fragmented in Australia, multiple organisations and settings were included: a large metropolitan public healthcare network, two community-based home care services and three general practices in the Australian city of Melbourne. To maximise sampling variation, healthcare practitioners from a range of disciplines were recruited from two inpatient sites in the public health network. In addition, participants were recruited from two community-based home care services focussed on community nursing and aged care, and general practice.

### Phase 1: Semi-structured interviews

#### Participants

Purposive sampling, using maximum variation for healthcare discipline and setting, was used to select participants for a semi-structured interview and included multidisciplinary practitioners across acute, sub-acute and community settings. To be included, healthcare practitioners needed to be employed by a participating organisation and providing transitional care to older adults and their carers. Management at each site was invited by the research team to contact health practitioners who provided transitional care to older adults in order to ascertain practitioners’ interest to learn more about the study. Interested health practitioners were sent a copy of the Participant Information and Consent Form, demographic questionnaire and interview guidelines by the research team. The first author explained the study and participation to interested practitioners, invited the practitioner to complete the demographic questionnaire to establish eligibility to participate, and a suitable time/date was scheduled for the interview.

Permission was requested from patients who had participated in an earlier study [24] for the first author to contact their general practitioner (family physician). The first author invited general practitioners to participate in the study and sent the Participant Information and Consent Form, demographic questionnaire and interview guidelines by email. The first author then contacted the general practitioner by telephone and explained participation in the study using the Participant Information and Consent Form and invited the practitioner to complete the demographic questionnaire to establish eligibility to participate. A time/date was scheduled for the interview.

#### Data collection tools

Data collection instruments included a demographic questionnaire and a semi-structured interview guide, including questions regarding participants’ experiences of providing transitional care: (1) What do you know about the transition of older adults from hospital to home? (2) What is the best thing that has happened in the transition of older adults from hospital to home? (3) What is the worst thing that has happened in the transition of older adults from hospital to home? Additional prompts included: What do you value in relation to transitional care? What is missing in transitional care?

The interview guide was informed by previous reviews [7,25], and recommendations from Bate and Robert [21]. Data were collected over a 12 month period from 2015–2016 including the period during which CDC was initiated in Australian community aged care [17]. From individual interviews, similar codes and categories emerged and saturation was deemed to be reached after 30 interviews. An additional 17 interviews were conducted across the range of settings and services to confirm data saturation.

#### Procedure and data collection

The first author conducted face-to-face semi-structured interviews in participants’ place of employment during a scheduled meal break or in an office at Deakin University. In all, 45 healthcare practitioners agreed to participate. Three general practitioners agreed to participate. Two general practitioners were interviewed face-to-face and one was interviewed by telephone. The semi-structured interview guide provided a framework for the interviews.

### Phase 2: Focus group

#### Participants

Participants were invited to take part in the focus group in a staff newsletter article introducing the study. Healthcare practitioners with a key role in transitional care at participating organisations were invited to participate. Patients and carers who had participated in another part of the study [24] were invited to take part in the focus group.

#### Data collection tools

The focus group interview guide was developed from guidelines provided by Bate and Robert [21]. In the focus group, a summary of the themes from the interview findings was presented and the questions ‘Do the findings make sense? How/how not?’ were used to guide the discussion. The focus group was conducted in April 2016.

#### Procedure and data collection

A total of seven participants took part in the focus group: a patient, a carer, one health provider who was also a carer, one healthcare practitioner from acute care, two from sub-acute care, and one from a community-based program. Participants who were interested to take part were sent the Participant Information and Consent Form and relevant details about the focus group date, time and location. Written consent to participate was sought at the focus group and demographic information was collected pertaining to participants’ age, gender, healthcare practitioner discipline and employing healthcare service. The first author conducted the focus group with the support of an assistant to note observations.

### Ethical considerations

Ethics approvals for Phases 1 and 2 of the study were obtained from the Human Research Ethics Committees at the participating healthcare services and Deakin University. Participants participated voluntarily in the study. Following an explanation of the study guided by the Participant Information and Consent Form, participants provided verbal and written consent. All data were de-identified. To preserve anonymity, identifying personal information was removed from transcripts.

### Member checking

The interview data for Phases 1 and 2 reflect the experience of the participant at the time and place of the interview. In accordance with a social constructivist standpoint, experience is contextualized by time and place [20,26]. The immediate time and place of the interview formed an important context that framed the data. Participants were not invited to check their transcripts because this would change the context of the interview. Themes derived from the semi-structured interviews were presented to focus group participants to support the consistency between the way participants and researchers perceived the data.

### Data sets

In both study phases, and with all participants’ permission, semi-structured interviews and the focus group interview were audio-recorded for transcription and data analysis. The first author transcribed the semi-structured interviews. A professional transcriber transcribed the focus group recording. For Phase 1, all semi-structured interview transcripts formed the principal data set for analysis. In Phase 2, one focus group interview transcript formed the principal data set for analysis.

### Data analysis

In study Phases 1 and 2, demographic information for participants was entered into Statistical Package for the Social Sciences (SPSS) version 21. Categorical data were analysed for frequencies, and continuous data were analysed using descriptive statistics. Qualitative data were analysed using inductive thematic analysis [27]. Using the research aim as a guide and supported by the Framework Approach, thematic analysis was an iterative process involving the comparing and contrasting of codes and categories within and between interviews and between participants [27,28]. All participants were allocated pseudonyms.

### Trustworthiness

The credibility of the study was supported by prolonged observation occurring over the two phases of the study [20,22,29,30]. Credibility is further supported by the triangulation of data from Phases 1 and 2 to give a multi-perspective description of integrated transitional care [20,22,29]. Transferability is enhanced through thick description of the study context, which was the focus of the semi-structured interviews conducted in Phase 1 of the study [20,22]. The study findings and inferences have been tested for consistency through dependability checks [22] where all authors coded a random sample of 6 complete raw data files and partial data files from remaining interviews. All authors interrogated interpretations made by the first author in regard to Phases 1 and 2 of the study over a series of 6 meetings by reviewing the detailed audit trail documented by the first author during data coding and analysis of data categories. Confirmability was supported through the first author’s use of field notes throughout data collection [29].

## Phase 1 Findings

Phase 1, involved 48 semi-structured interviews with healthcare practitioners from a range of disciplines working in one acute medical ward, one sub-acute rehabilitation ward, two community-based organisations and three general practices. Study participants were on average aged 44 years (SD 11.6 years, range 23 to 64 years), 40 (83.3%) were female and most participants (n = 38, 79.2%) spoke English at home. Study participants were from nursing, medicine and allied health, and were employed across a range of in-patient and community settings and programs with an acute, sub-acute or community-based care focus. In total, 48 practitioners participated in a semi-structured interview. Additional demographic information is presented in Table 1.

**Table 1:** Demographic Characteristics of Healthcare Practitioners (n = 48).

Demographic information	Frequency (%)

Country of birth	
Australia	37 (77.1)
UK	2 (4.2)
India	2 (4.2)
Other	7 (14.5)
Highest qualification	
Diploma (TAFE)	3 (6.3)
Bachelor	31 (64.6)
Graduate Certificate	6 (12.5)
Graduate Diploma or Master’s	8 (16.7)
Discipline	
Registered nurse	25 (52.1)
Social worker	8 (16.7)
Medical practitioner	7 (14.6)
Physiotherapist or occupational therapist	4 (8.4)
Enrolled nurse	2 (4.2)
Other allied health	2 (4.2)
Employer	
Large public health network	33 (68.8)
Community nursing service	7 (14.6)
Aged care case management service	6 (12.5)
Private practice	2 (4.2)
Current role	
Registered nurse	23 (47.8)
Case manager	8 (16.7)
Allied health practitioner	8 (16.7)
Medical practitioner	7 (14.6)
Enrolled nurse (licensed to practice nurse)	2 (4.2)
Participants by ward/program	
Acute medical ward	12 (25.0)
Hospital in the Home	2 (4.3)
Geriatric Evaluation and Management (rehabilitation) (GEM) ward	8 (16.7)
Aged care consultancy and triage team	3 (6.2)
Post-Acute Care (PAC)	4(8.3)
Transition Care Program (TCP)	3(6.2)
Aged care case management	6 (12.5)
District nursing	7 (14.6)
General practice	3 (6.2)

Participants described their experience of providing integrated transitional care to older adults and carers from hospital to home in terms of four main themes: 1) Rapid and safe care transition; 2) Discussing as a team; 3) Questioning the discharge; and 4) Engaging patients and carers. Participants in inpatient settings and general practice perceived that community-based healthcare professionals were accessible to older patients to support their care transitions. Most participants were unaware of reforms in community aged care and the introduction of the CDC model.

### Rapid and safe care transition

All participants in all settings described their experience of providing transitional care in terms of the main theme ‘Rapid and safe care transition’. They explained this further in three sub-themes: 1) Discharging early: transitioning to the right place; 2) Communicating against the system; and 3) Re-admitting early.

All participants in the inpatient acute and rehabilitation wards reported that their aim was to maximise bed availability, and prevent harm. They perceived that due to the efficiency pressures, there was a need to discharge patients from the ward as quickly as possible to the most appropriate destination. They explained that when patients were too sick to be discharged from the acute ward, the senior nurse needed to defend this decision with management.

“So on days when there are lots of unwell patients, who are medically unstable, you might have one discharge. And you have Access on the phone saying, ‘Come on, who else is going home?’ No one is going home, they are all sick, you can’t send them home!” (Maria, nurse, acute care)

Most inpatient participants explained that it was not always possible for all involved parties to communicate about transitional care. They said that it was particularly difficult to communicate effectively with community-based practitioners and carers.

“Discharge planning can go astray. Because there is a disconnect between acute care, general practitioners, primary care, carers, the whole gamut. We don’t have one communication tool that everybody can look into and be aware of.” (Rachel, nurse, sub-acute care)

Most participants explained that early re-admission following transition home was one of the worst outcomes. According to participants, the push for hospital beds could result in problems with transitional care due to insufficient time and resources to conduct assessment and care planning.

“When the patient actually ends up back in again. And you think that the patient is doing pretty well and will be able to perform well at home again. This is disappointing and it’s a bit depressing as well.” (Joanne, nurse, acute care)

### Discussing as a team

Most participants in acute and sub-acute settings described multidisciplinary team discussion as a solution to ‘Rapid and safe care transition’. Discussing as a team was characterised by two subthemes: 1) Raising concerns, and 2) Putting it all together.

According to participants in acute and sub-acute care, problem solving care transitions for adults with complex care needs involved multidisciplinary team collaboration. They considered that regular multidisciplinary team meetings in acute and sub-acute care programs and wards facilitated integrated transitional care because different perspectives from different disciplines were voiced and considered.

“So we go through each patient with the registrar that outlines the medical plan and we discuss as a team. What the plan is as well. … We raise concerns that we have, we raise the patient’s perspective, what the patient wants. And then it’s discussed as a team what the plan is.” (Margaret, allied health, acute care)

Some participants in acute and sub-acute care, perceived that although practitioners valued and listened to comments from each member of the multidisciplinary team, they could also have discrepant views and practitioners could assert different opinions about the focus of the transitional care plan.

“… once everyone got together most of the time it could get quite fiery. The doctors would see something completely differently from what allied health would see. Allied health would be like they’ve come in three times in the past. And the medical team well, their white cell markers are fine so we’re happy for them to go. We’re like No! It can get quite heated.” (Rowena, nurse, acute care)

Many participants commented that practitioners needed to engage in team discussion in order to reconcile their discipline-specific assessments against those of other disciplines.

“Most times I think we get it right, because you are not making your decisions solely on the basis of your nursing assessment. You get your OT, your physiotherapist, your social work, your medical assessments happening as well. But the process is very quick and I don’t necessarily think that the acute care environment allows the patient the time to really declare their true abilities.” (Amy, nurse, sub-acute care)

Participants in community and general practice, and some participants in acute and sub-acute care noted there was a need for improved collaborative assessment about aged care issues.

### Questioning the hospital discharge

All participants in community-based services and some participants in sub-acute care programs explained how they questioned the hospital discharge plan in order to ensure that it was effective in the home environment. This theme was characterised by two sub-themes: 1) Linking with the hospital; 2) Being in their own environment.

All community nurses noted their point of contact was the community nursing liaison service co-located in the health network. They perceived that the liaison community nurses advocated and negotiated transitional care planning with the multidisciplinary teams.

“Liaison for [community nurses] is like the representative from [the service] to make sure that all of the discharge needs are provided. And that’s our person that we can go to if it’s been a client who has gone back to hospital and we want to try and influence the discharge.” (Joan, allied health, community nursing organisation)

Many aged care case manager participants commented that they used the strategy of finding one health practitioner in the in-patient setting as a point of contact to have some influence in the discharge plan.

“I’ll find a point of contact and make that one person my point of contact…Whether that is the head nurse or social worker.” (Sally, case manager, community aged care)

All community-based participants explained that they needed to question the follow-up components of the transitional care plan and re-assess the person at home during a home visit because the person’s care needs had changed. In contrast to many ward-based participants, all community-based participants perceived that when the person was ready for hospital discharge, this meant that they were also functionally safe at home.

“A man recently sent home from hospital was on insulin and he was sent home with three different types of insulin, he had no idea which one he was on…He didn’t have any idea how to work his blood sugar machine. He didn’t know anything about what to do if he had a hypo [hypoglycaemic episode]. I mean supposedly the diabetic educator (sic) had seen him. But when I saw him he was quite muddled. And he didn’t appear to have knowledge or confidence.” (Anna, nurse, community)

### Engaging patients and carers

Most participants explained that they engaged patients and carers in transitional care in response to the problem of ‘Rapid and safe care transition’. They explained this theme in three sub-themes: 1) Considering patients’ wants and needs; 2) Family are an amazing resource; and 3) Maintaining independence.

Most participants reported that they considered their patients’ wishes and needs in transitional care by discussing the patient’s goals, expectations and concerns. They commented that they needed to listen, be empathic, and respect that the person’s and family’s/carers’ wishes could be different from those of the healthcare practitioners.

“We consider their wants and their needs and we always think about the patient first and their family. We respect what they might like and if we have an opinion that they might not be safe to return home, but they are adamant that they want to return home, we’ll facilitate that the best way that we can.” (Michelle, allied health, acute care)

Some participants commented on difficulty considering patient and family wishes given restrictions in resources.

“It is really important to let them know right from the beginning that when our program finishes I cannot guarantee ongoing services. And that is really challenging because you can see in people’s faces ‘what are we going to do’?” (Melanie, case manager, community-based sub-acute care)

According to most participants, family and carers were the main supports for the person and it was essential to engage them in discussion about transitional care.

“Family are an amazing resource and our first go-to along with the general practitioner in terms of collateral history… The family members are often the ones making the decisions on their behalf, either formally or informally.” (David, medical practitioner, aged care consultancy)

Many participants reported that their patients wanted to be at home and it was therefore important to discuss self-care education, support their return to independence and minimise actions that could limit independence. Deanna, allied health from acute care, commented on the need to support patients’ independence at home in regard to medications:

“You don’t want to take away someone’s independence either… So take away the responsibility of managing their own medications and put them on a [dose administration container] they start losing touch with what they are actually taking and then they are not engaged anymore.”

**Figure 1:**
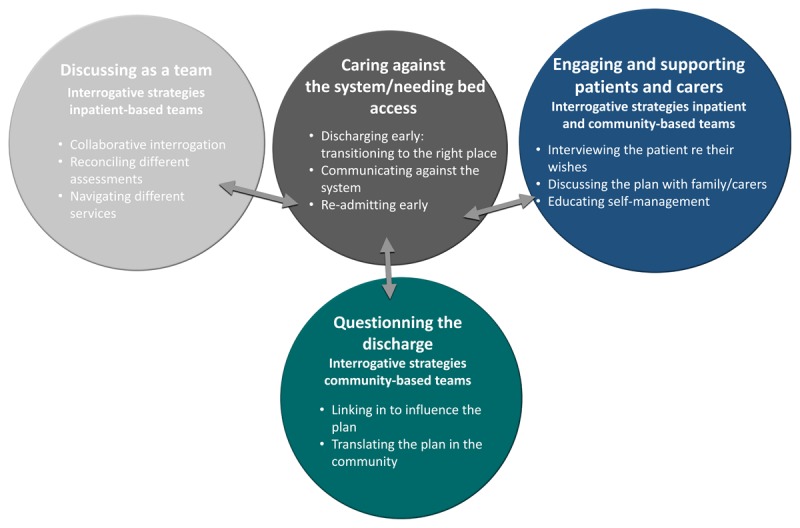
Model of interrogative social processes in integrated transitional care.

Findings from the semi structured interviews are presented in Figure 1.

## Phase 2 Findings

In Phase 2, one focus group was conducted with seven participants including five practitioners. One patient and one informal caregiver also participated in the focus group. Participants were aged 52 years on average (range 27 to 76 years). Most participants were female (6) and most were born in Australia (5). Participant healthcare practitioner were acute, sub-acute and community settings. One practitioner was also a carer. Following analysis of the transcript data, two main themes were identified: 1) Needing bed access; and 2) Service navigation. Participants reported agreeing with the interview findings.

All participants referred to ‘needing bed access’ as the major problem that required addressing in transitional care. In acute care, the goals were to ensure the efficient flow of patients through the healthcare system, safe discharge and the avoidance of early readmission.

“Within four hours we have got to have patients in and out of emergency departments. That could be back home or up into the wards. So everything we do within the acute environment now is very much focused on trying to meet those targets, get patient flowthrough.” (Catherine, a healthcare practitioner in sub-acute care)

According to participants, there was limited time to discuss and communicate with community-based practitioners regarding important information about the person and this increased the risk of early readmission to hospital. Several participants from sub-acute and acute care settings explained that healthcare practitioners used a range of service navigation strategies in response to needing bed access. These strategies included assessment; discussion and coordination by multidisciplinary and aged care teams, and engagement and education of patients and carers.

## Discussion

Findings from the current study indicate strengths and weaknesses in integrated transitional care for older adults in an Australian setting. Strengths include the range of communication processes used by participant healthcare practitioners across settings and programs to optimise care integration and successful care transitions. The main weakness identified was the limited understanding of pending changes in community-based aged care to a CDC model, and the related increase in responsibility for older adults and their informal carers in their own transitional care.

Findings from Phases 1 and 2 of the study suggest that according to study participants the fundamental social problem to be solved in transitional care of older adults from hospital to home is a systems problem of early discharge and fragmented services. This is a common finding across many other studies in transitional care [13].

Findings from Phases 1 and 2 of the current study further indicate that healthcare practitioner participants perceived that the social processes of interrogation, including discussion, questioning and patient engagement, were essential to effective communication and coordination of care to navigate transitions across settings and disciplines. In developing the discharge plan, health practitioners in the inpatient setting discussed as a team to raise concerns and to reconcile differences. Practitioners outside the healthcare network including those in the community questioned the hospital discharge plan through their use of questions to influence the plan and also to translate the plan into the home. Across settings and services, practitioners engaged patients and carers to establish goals, build relationships, discuss the plan and provide education. Participants reported that they engaged in patient education with an expectation that healthcare practitioners would be accessible to patients at follow-up.

Transitional care has been identified as an important clinical process that indicates the quality of care integration across services and settings [13,14]. Previous studies have also found that communication and care coordination were central to the effectiveness of transitional care [1,2,31]. Recent research by Valentijn et al. [9] and Banfield et al. [8] introduces a taxonomic understanding of integrated care in primary care contexts. Findings from the current study expand on this research by highlighting communication and care coordination processes of discussion, questioning and patient engagement that are employed by practitioners at the clinical care level. Although practitioners in different settings adopted similar communication and coordination processes, they used different strategies to navigate care across settings. The strategies were dependent upon the systems-based barriers they encountered in either inpatient or community settings. This indicates that practitioners need to use and apply communication and coordination skills as part of an adaptive problem solving process during older adults’ care transitions.

Findings from the current study and from previous research emphasise that future implementation initiatives in transitional care must focus on supporting communication processes of discussion, questioning and patient engagement, between practitioners in all settings and programs, and with patients and informal carers. However, this is complicated in the Australian context by recent changes in community-based aged care.

In accordance with community expectations for greater consumer choice and control in their health and aged care, in 2015 the Australian federal government undertook substantial reforms in community aged care including investment in consumer-directed models of community-aged care [17,32,33]. Concurrently, discharged patients were required to self-refer for long-term aged care in the community, and some publicly funded models of community care including professional district nursing were de-funded [17]. This occurred without substantial funding increases for service navigation, professional nursing or allied health in general practice. Notwithstanding the many benefits of CDC for older adults and their informal carers, the shift to CDC has increased responsibility for older people and their informal carers in their own care, including transitional care [17]. Yet, findings from the current study indicate that most participant healthcare practitioners in the inpatient setting and in general practice assumed that follow-on professional support would be available to their patients following discharge. Few participants were aware of the reforms and implementation of CDC and the associated increase in responsibility for patients and informal carers in transitional care or additional supports that they might require to negotiate and navigate their own care transition. Participants in inpatient care and general practice did not explain any planned changes in assessment of older adults in relation to self-care abilities including those related to discharge, service navigation and care transition back to the community.

### Further research

Further research is warranted to determine how to best support healthcare professionals in assisting patients and informal carers to negotiate and navigate their transitional care. This may include the development and implementation of tools and communication aids to support assessment of self-management in relation to chronic disease. New roles and models for healthcare practitioners may require development and evaluation in inpatient and community settings such as general practice to support service navigation for older adults and their informal carers experiencing care transitions.

### Study strengths and limitations

The research comprised semi-structured interviews, which supported a detailed and rich description of participants’ experiences of provision of transitional care to older adults. Because participants were purposively selected, it cannot be claimed that findings represent the experiences of all healthcare practitioners. Data saturation would suggest the themes were frequent experiences among multidisciplinary and multi-site participants. The study included participants in in-patient acute medicine, rehabilitation, and community care programs. Application and transfer of findings to other programs, such as surgical services, cannot be claimed. However, our findings may have transferability to similar contexts of care elsewhere.

## Conclusions

The findings from this Australian study have implications for transitional care. Healthcare practitioners use a range of discussion, questioning and patient engagement processes to communicate with each other and with patients and carers, and to coordinate transitional care. In Australia, communication skills are part of the educational preparation and continuing education of healthcare practitioners, however the degree to which this education is focussed on improving negotiation and navigation of care transitions for and with older adults is unclear. Most participants were unaware of pending changes related to the implementation of a CDC program in community-based aged care. Findings from the study highlight the need for health practitioners to adapt their care coordination and communication practice to an evolving care context of stronger expectations that older adults and their informal carers will take greater responsibility for their own care in the community. In care transition contexts shaped by multidisciplinary teams, sub-acute care and consumer-directed care, health practitioners should focus on supporting older adults and their informal caregivers to navigate their own care transitions. To improve care integration during older adults’ care transitions, health services organisations and planners should adapt systems to support health practitioners in assessment of patients’ self-care abilities regarding negotiation and navigation of their own care transitions.
